# Alcohol amplifies ketamine-induced apoptosis in primary cultured cortical neurons and PC12 cells through down-regulating CREB-related signaling pathways

**DOI:** 10.1038/s41598-017-10868-z

**Published:** 2017-09-05

**Authors:** Daiying Zuo, Feng Sun, Jiahui Cui, Yumiao Liu, Zi Liu, Xuejiao Zhou, Zengqiang Li, Yingliang Wu

**Affiliations:** Department of pharmacology, Shenyang Pharmaceutical University, 103 Wenhua Road, Shenyang, Liaoning 110016 P.R. China

## Abstract

Recreational use of ketamine (KET) has been increasing worldwide. Previous studies have demonstrated that KET induced neurotoxicity; however, few studies have examined how alcohol (ALC) affects KET-induced neurotoxicity. In light of the fact that some KET abusers combine KET with ALC, the present study was aimed to investigate the effects of ALC on KET-induced neurotoxicity and the underlying mechanism *in vitro*. Our data revealed that co-treatment with ALC and KET was more detrimental to cell viability than KET single treatment in both PC12 cells and primary cultured rat cortical neurons. Furthermore, ALC exacerbated KET-induced apoptosis characterized by morphological changes and the sub-G1 phase increase, which were mitigated by the pretreatment of CNQX, a known alpha-amino-3-hydroxy-5-methyl-4-isoxazole propionate (AMPA)/kainite (KA) receptor antagonist. In addition, ALC and KET co-treatment led to intracellular Ca^2+^ overload, down-regulation of p-Akt, p-CREB, PKA, CaMK-IV, Bcl-2 and BDNF expression and up-regulation of cleaved caspase-3 and Bax expression, which can be attenuated by CNQX pretreatment. These results indicate that the potentiation of ALC on KET-induced neurotoxicity was related to the down-regulation of CREB-related pathways. Our present study also indicates that ALC and KET co-abuse might cause serious neurotoxicity which should be conveyed to the public and drew enough attention.

## Introduction

Glutamatergic receptors can be classified as ionotropic (iGluRs) and metabotropic (mGluRs) receptors. For the subtype of iGluRs, there are N-methyl-D-aspartate (NMDA), alpha-amino-3-hydroxy-5-methyl-4-isoxazole propionate (AMPA) and kainite (KA) receptors^1, 2^. Ketamine (KET), also known as “special K”, is an NMDA receptor antagonist used in infants and toddlers as a dissociative anesthetic for surgery because of its favorable safety profile. Unfortunately, our previous studies and others have shown that KET has dose-dependent neurotoxicity *in vivo* and *in vitro*, especially when being used during the periods of brain growth^3–10^. In addition, KET is a mainstream recreational substance with increasing prevalence of illegal abuse, especially in adolescents^11^. It is most commonly abused as a “club drug” at bars, clubs, concerts, and parties because of its strong hallucinogenic effects, amplified sensations and an escape from reality. It was reported that KET exposure causes brain microstructural abnormalities in adolescent monkeys^12^. Therefore, KET abuse-induced neurotoxicity needs to be addressed.

Alcohol (ALC) is a psychoactive substance widely consumed by the society. Due to its low molecular weight and water solubility, ALC can be distributed to almost all tissues, including being able to cross the blood–brain barrier (BBB) and reaching the central nervous system (CNS), which can cause neurotoxic effect^13, 14^. It was reported that ALC intoxication resulted in neuronal death, balance impairment, behavior changes and altered body movement coordination^15–17^. Furthermore, ALC abuse has been shown to cause aberrations in synaptic plasticity and related neuronal function^18^. In fact, some KET consumers have been reported to combine KET with ALC. There is evidence for mixed-drug abuse intoxications involving KET and ALC^19, 20^. In these cases, KET intoxication was considered as the main reason of death. Nevertheless, in our opinion, further concerns should be considered regarding the combination of ALC and KET, as there is a possible risk of synergistic interaction between KET and ALC that may play a vital role in the mixed-drug intoxications, especially the neurotoxicity. However, there have been limited studies on the neurotoxicity of the combination of ALC and KET. Thus, investigating the consequence of this combination remains important, since a synergism of their effects may increase the neurotoxicity.

PC12 cells, a rat pheochromocytoma cell line, differentiate both morphologically and biochemically into nerve cells under the treatment of nerve growth factor^21^. Thus, PC12 cells have been widely accepted as a model for investigating neuronal properties and neurotoxicity *in vitro* ^22, 23^. In light of the emerging combined use of ALC and KET, the present investigation was undertaken to study the role of ALC in KET-induced neurotoxicity by comparing the effects of KET alone or combined treatment with ALC in PC12 cells. Furthermore, primary cultured neurons were used to further prove the effect of ALC on KET-induced neurotoxicity. 6-cyano-7-nitroquinoxaline-2,3-dione (CNQX), an AMPA/KA receptor antagonist, was used to study the involvement of AMPA/KA receptors on ALC and KET- induced neurotoxicity. In addition, the pathways that are essential for cell survival such as the activation of Akt (a serine/threonine kinase or protein kinase, PKB), protein kinase A (PKA), cyclic AMP-responsive element binding protein (CREB), brain-derived neurotrophic factor (BDNF) and apoptosis-related proteins, were further investigated to explore the underlying molecular mechanisms of ALC and KET-induced neurotoxicity in PC12 cells.

## Results

### ALC potentiates KET-induced cell viability decrease, ROS production and morphological changes in PC12 cells

**Figure 1:**
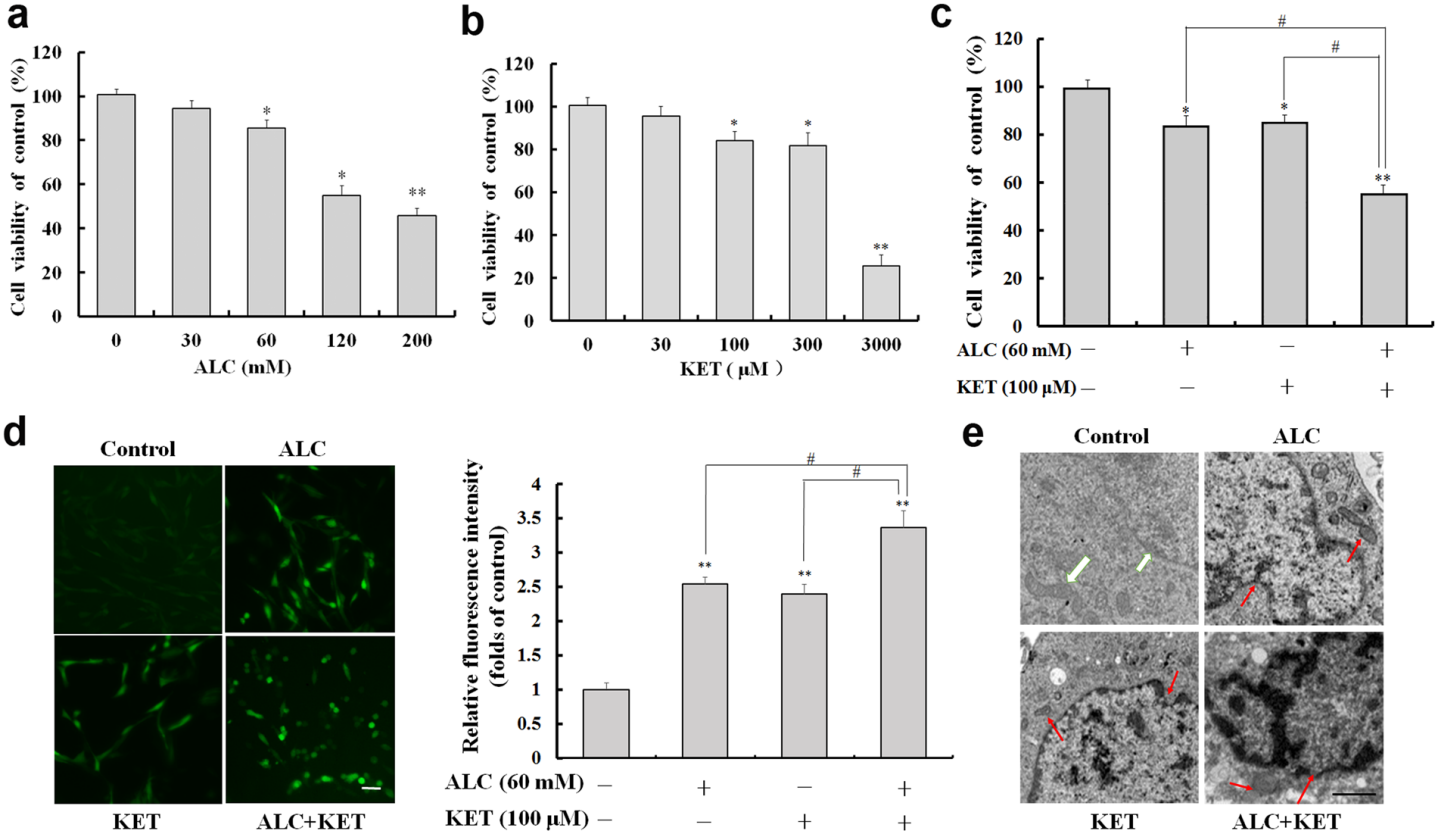
Cell viability, ROS level and morphological changes induced by ALC and/or KET in PC12 cells. (**a**) ALC (60–200 mM) dose-dependently decreased the cell viability of PC12 cells. (**b**) KET (100–3000 μM) dose-dependently decreased the cell viability of PC12 cells. (**c**) ALC (60 mM) potentiated KET (100 μM)-induced cell viability decrease in PC12 cells. (**d**) ALC (60 mM) potentiated KET (100 μM)-induced ROS level increase in PC12 cells by DCFH-DA staining. Scale bar = 20 μm. (**e**) ALC (60 mM) potentiated KET (100 μM)-induced morphological changes in PC12 cells by transmission electron microscope. Scale bar = 1 μm. The white arrows represent cells with normal nuclei and mitochondria. The red arrows represent abnormal nuclei and mitochondria. *p < 0.05, **p < 0.01 compared with the control group. ^#^ *p < *0.05 compared with the group treated with ALC and KET.

As shown in Fig. 1a, ALC treatment at 30 mM for 24 h had no cytotoxic effect on PC12 cells by the 3-(4,5-dimethylthiazol-2-yl)-2,5-diphenyltetrazolium bromide (MTT) assay. The higher doses of ALC (60–200 mM) treatment for 24 h significantly decreased cell viability of PC12 cells in a dose-dependent manner. KET treatment at 30 μM for 24 h did not significantly influence the cell viability of PC12 cells. The higher doses of KET (100–3000 μM) treatment for 24 h dose-dependently decreased the cell viability of PC12 cells (Fig. 1b). Based on the above data, further experiments were conducted to study the effect of ALC (60 mM) and KET (100 μM) co-exposure for 24 h on the cell viability of PC12 cells. As shown in Fig. 1c, the co-exposure of ALC and KET showed a synergistic effect on the loss of cell viability compared with the individual exposure to ALC or KET at the same doses (*p* < 0.05). Moreover, the levels of reactive oxygen species (ROS) in PC12 cells were investigated by 2′,7′-dichlorodihydrofluorescein diacetate (DCFH-DA) staining. As shown in Fig. 1d, the ROS levels reflected by fluorescence intensity in the ALC (60 mM) or KET (100 μM) alone treatment groups were significantly increased compared with that of the control group (*p* < 0.01). ALC (60 mM) and KET (100 μM) co-exposure for 24 h further increased the levels of ROS compared with the ALC or KET alone treatment groups (*p* < 0.05). Furthermore, transmission electron microscopy was used to study the ultrastructural changes after treatment with ALC and/or KET. As shown in Fig. 1e, the nuclear membrane was smooth with normal chromatin, and the mitochondrial membrane was smooth with normal mitochondrial cristae in the control group. ALC (60 mM) or KET (100 μM) alone treatment for 24 h led to chromatin margination and decreased mitochondrial cristae, which were more obvious when cells were co-exposed with ALC (60 mM) and KET (100 μM) for 24 h.

### KET-induced cell viability decrease in primary cultured neuronal cells is potentiated by ALC

**Figure 2:**
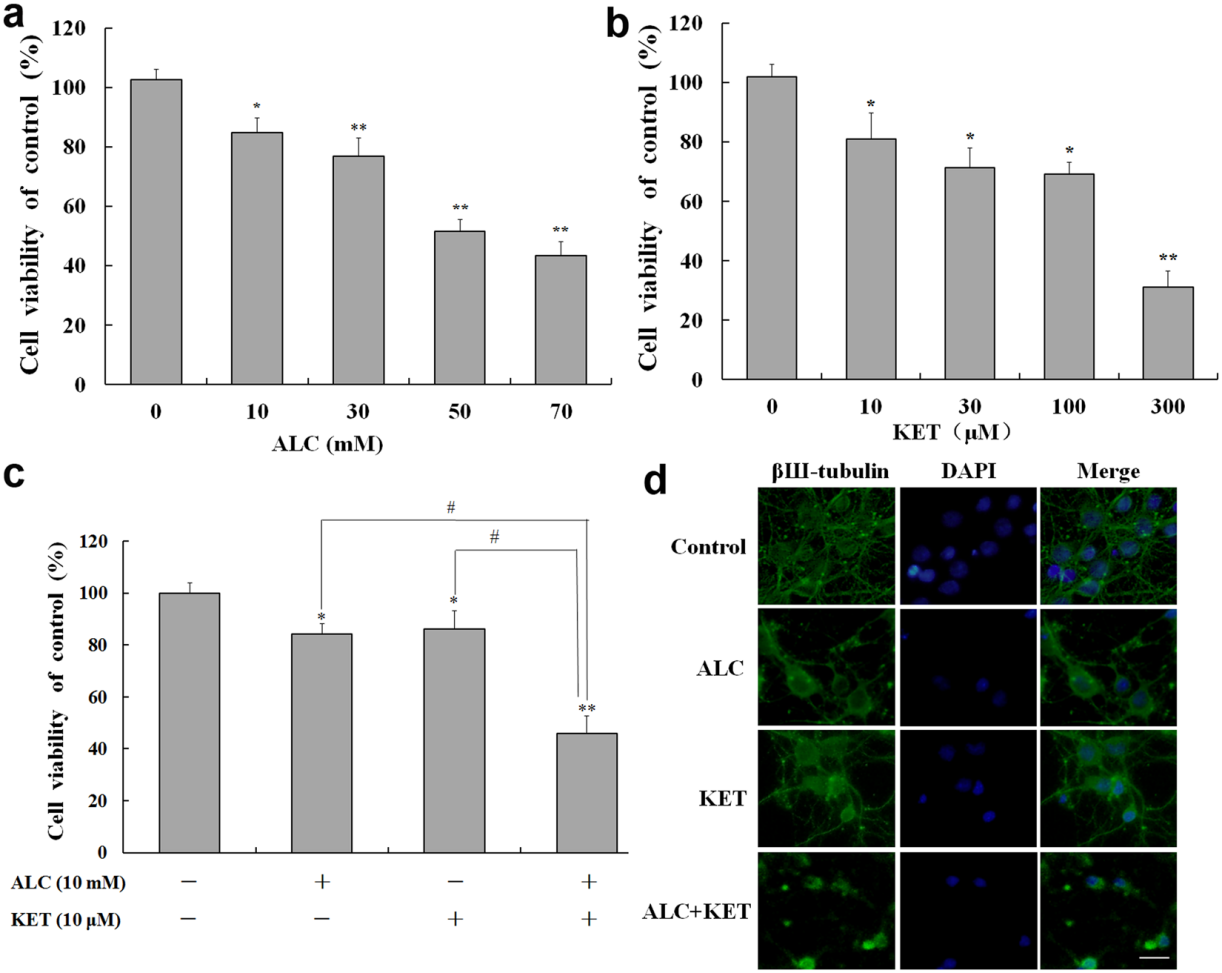
Cell viability assessed by MTT assay and morphological change assessed by immunofluorescence staining in primary cultured cortical neurons. (**a**) ALC (10–70 mM) dose-dependently decreased the cell viability of neurons. (**b**) KET (10–300 μM) dose-dependently decreased the cell viability of neurons. (**c**) ALC (10 mM) potentiated KET (10 μM)-induced cell viability decrease of neurons. (**d**) ALC (10 mM) potentiated KET (10 μM)-induced morphological change assessed by immunofluorescence staining using βШ-tubulin and DAPI. Scale bar = 20 μm. *p < 0.05, **p < 0.01 compared with the control group. ^#^ *p < *0.05 compared with the group treated with ALC and KET.

Primary cultured neuronal cells of the cortex were used to further test the effect of ALC on KET-induced neurotoxicity. As shown in Fig. 2, ALC (10–70 mM, Fig. 2a) or KET (10–300 μM, Fig. 2b) treatment for 24 h dose-dependently decreased the cell viability of neurons. Based on the above data, further experiments were conducted to study the effect of ALC (10 mM) and KET (10 μM) co-exposure for 24 h on the cell viability of primary cultured neuronal cells. The different doses were used between PC12 cells and primary cultured neuronal cells because different cell types have various sensitivities to ALC or KET. As shown in Fig. 2c, ALC (10 mM) and KET (10 μM) co-treatment for 24 h significantly decreased neuronal viability compared with ALC or KET treatment alone at the same dose (*p* < 0.05). Figure 2d shows the morphological changes using βШ-tubulin and 4′, 6-diamidino-2-phenylindole (DAPI) staining. The neurons in the control group grew well with normal neuronal soma and synapses. ALC (10 mM) or KET (10 μM) treatment for 24 h decreased the number of neurons and disrupted neuronal soma and synapses, which can be found severer with co-exposure of ALC (10 mM) and KET (10 μM) for 24 h to the cells.

### ALC potentiates KET-induced decrease in cell viability and the involvement of AMPA/KA receptors

**Figure 3:**
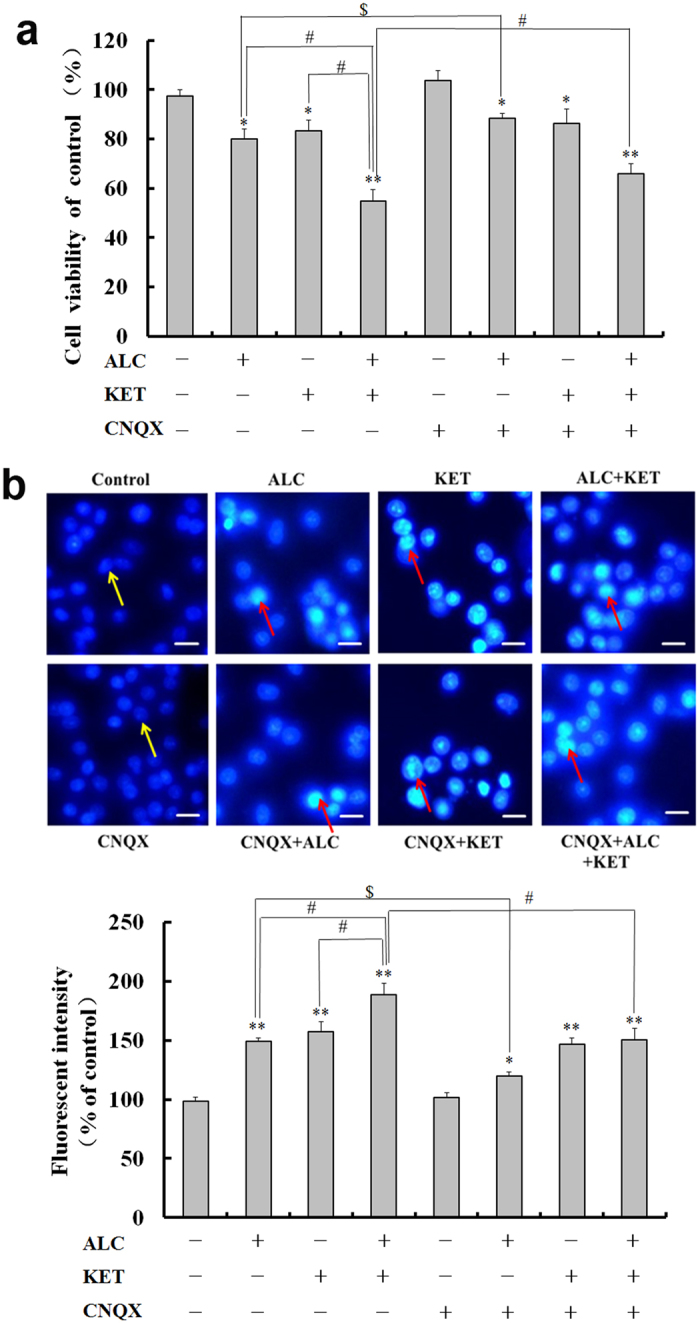
The cell viability decrease and morphological change induced by ALC and KET are attenuated by CNQX pretreatment. (**a**) CNQX (50 μM) pretreatment significantly attenuated the decrease of cell viability of PC12 cells induced by ALC or ALC + KET. (**b**) CNQX (50 μM) pretreatment attenuated the morphological change of nucleus induced by ALC and KET. The yellow arrows represent normal nuclei in the control group. The red arrows represent nuclear chromatin aggregation and apoptotic bodies after ALC and/or KET treatment. Scale bar = 20 μm. *p < 0.05, **p < 0.01 compared with the control group. ^#^ *p < *0.05 compared with the group treated with ALC and KET. ^$^ *p < *0.05 compared with the group treated with ALC.

The AMPA/KA receptor inhibitor CNQX was used to confirm if AMPA/KA receptors are involved in the cytotoxicity of ALC and KET. The dose selection of CNQX was based on the reference^24^ and our preliminary experiments. As shown in Fig. 3a, CNQX (50 μM) pretreatment significantly attenuated the decrease in cell viability of PC12 cells induced by ALC or ALC + KET. However, KET-induced decrease in cell viability was not significantly altered by CNQX pretreatment. Furthermore, we tested the morphological change of the nucleus by DAPI staining. As shown in Fig. 3b, cells being treated with ALC and/or KET for 24 h exhibited nuclear chromatin aggregation and apoptotic bodies. CNQX pretreatment could mitigate these morphological changes of nucleus induced by ALC or ALC + KET, which was consistent with the MTT results in Fig. 3a.

### ALC potentiates KET-induced apoptosis

**Figure 4:**
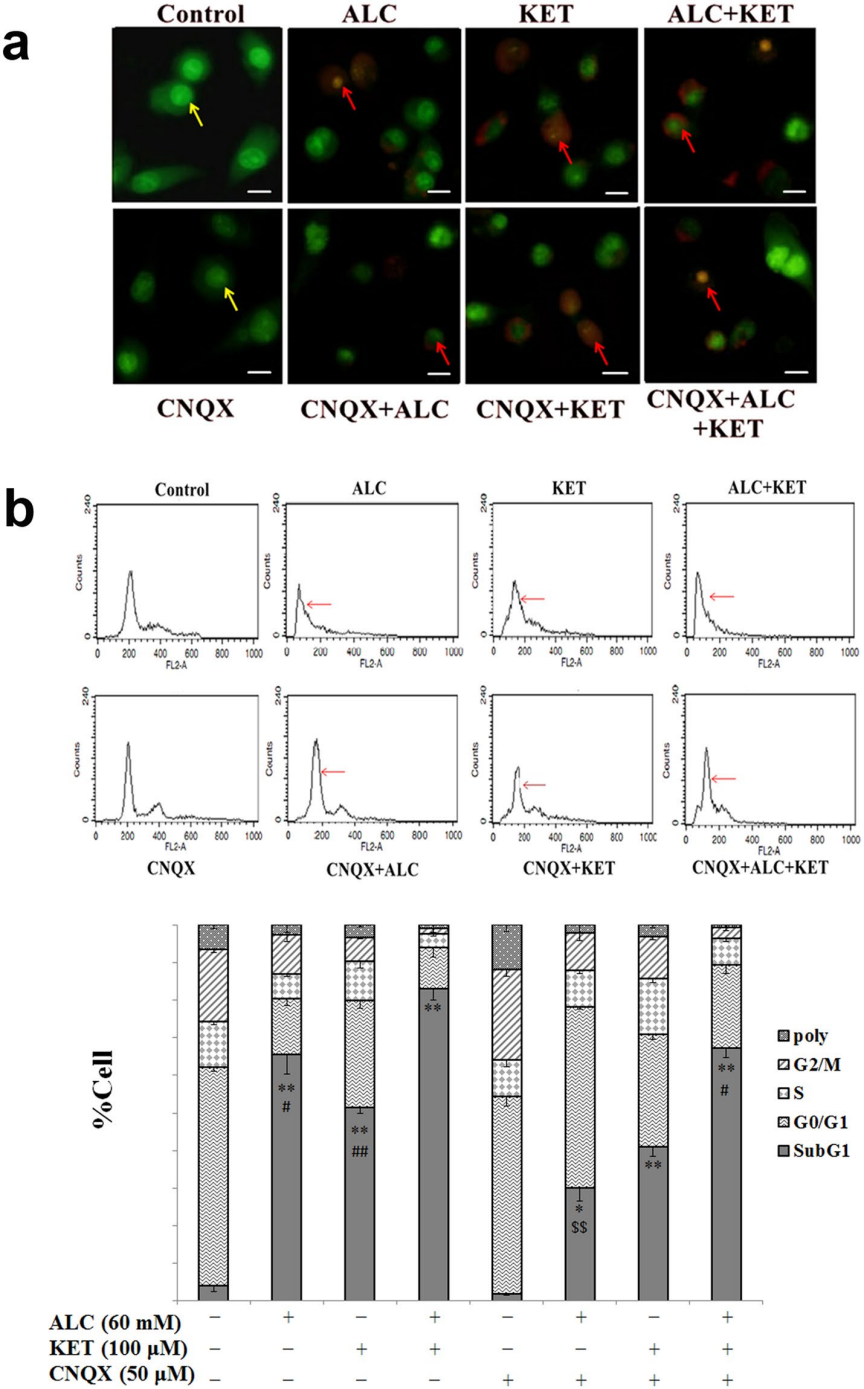
ALC potentiates KET-induced apoptosis. (**a**) ALC (60 mM) potentiated KET (100 μM)-induced apoptotic changes investigated by AO-EB staining. The yellow arrows represent normal nuclei in the control group. The red arrows represent apoptotic changes after ALC and/or KET treatment. Scale bar = 20 μm. (**b**) ALC (60 mM) potentiated KET (100 μM)-induced sub-G1 phase increase investigated by flow cytometry analysis. *p < 0.05, **p < 0.01 compared with the control group. ^#^ *p < *0.05, ^##^ *p < *0.01 compared with the group treated with ALC and KET. ^$$^ *p < *0.01 compared with the group treated with ALC.

To further confirm if ALC potentiates KET-induced apoptosis in PC12 cells, acridine orange-ethidium bromide (AO-EB) staining and flow cytometry analysis were performed. As shown in Fig. 4a, the nuclei of PC12 cells in the control group were in normal shape and stained with AO in green. ALC or KET treatment for 24 h induced apoptotic changes characterized by increased nuclei stained with EB in orange and nuclear chromatin aggregation, which was more significant when ALC and KET co-exposure for 24 h to the PC12 cells. CNQX (50 μM) pretreatment could notably attenuate the apoptotic changes induced by ALC or ALC + KET. Furthermore, as shown in Fig. 4b, KET-induced apoptosis which was characterized as an increase in sub-G1 phase, was significantly enhanced by ALC treatment. CNQX pretreatment could significantly decrease ALC- and ALC + KET-induced sub-G1 phase increase (*p* < 0.05); however, there was no significant effect on KET-induced sub-G1 phase increase.

### ALC potentiates KET-induced increase of intracellular calcium [Ca^2+^]_i_ levels

**Figure 5:**
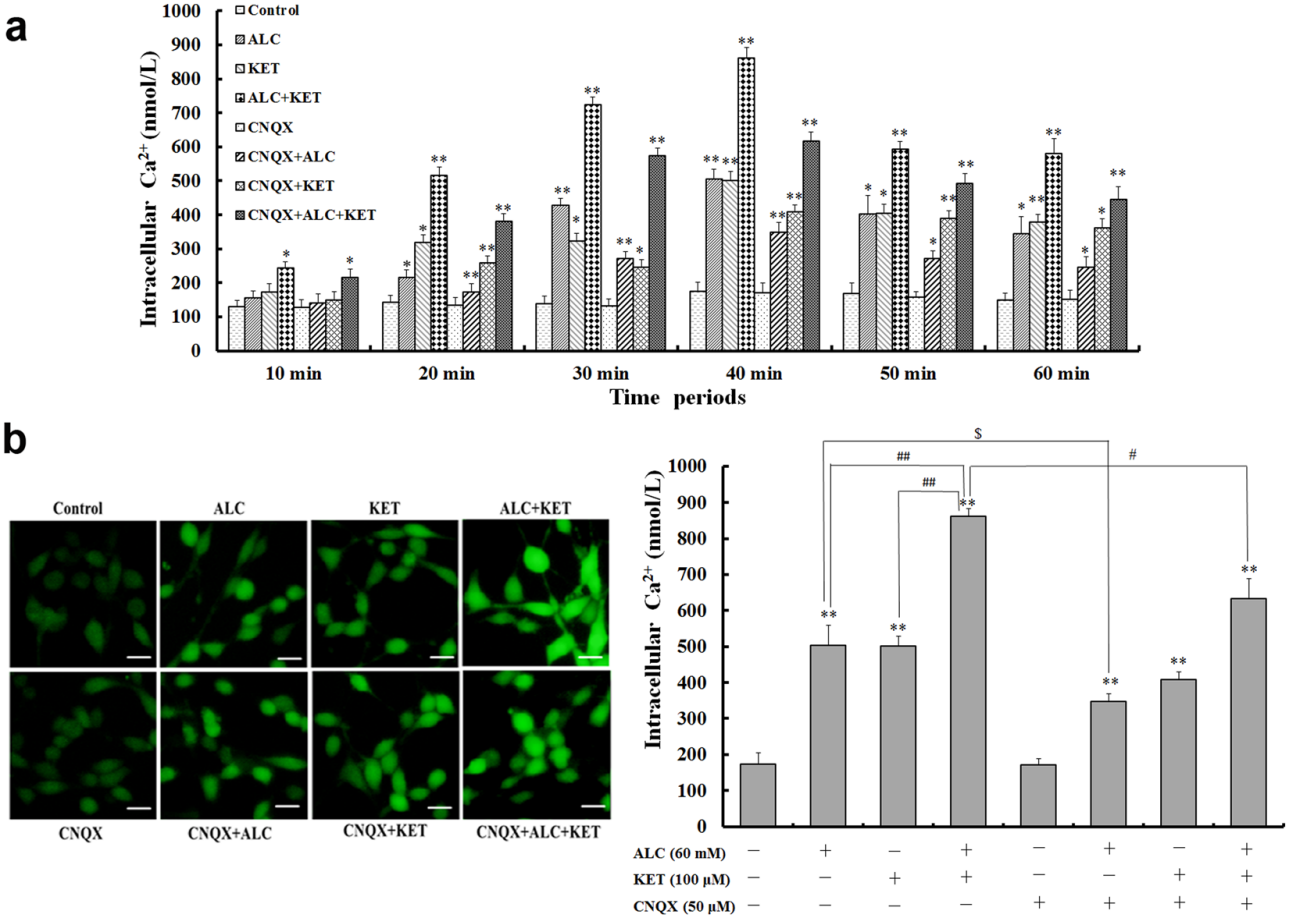
ALC potentiates KET-induced [Ca^2+^]_i_ levels. (**a**) ALC (60 mM) and/or KET (100 μM) treatment time-dependently increased intracellular Ca^2+^ levels of PC12 cells. The maximal [Ca^2+^]_i_ levels were achieved at 40 min after ALC and/or KET treatment. (**b**) ALC (60 mM) and KET co-treatment significantly increased [Ca^2+^]_i_ levels at 40 min compared with ALC or KET treatment alone. CNQX (50 μM) pretreatment attenuated ALC and ALC + KET induced [Ca^2+^]_i_ level increase. Scale bar = 20 μm. *p < 0.05, **p < 0.01 compared with the control group. ^#^ *p < *0.05, ^##^ *p < *0.01 compared with the group treated with ALC and KET. ^$^ *p < *0.05 compared with the group treated with ALC.

AMPA/KA receptor activation may increase Ca^2+^ influx. As a result, we further explore the [Ca^2+^]_i_ levels after ALC and/or KET treatment for different times. As shown in Fig. 5a, ALC (60 mM) or KET (100 μM) treatment could time-dependently increase [Ca^2+^]_i_ levels of PC12 cells. The increase of [Ca^2+^]_i_ levels was more significant after ALC and KET co-treatment than ALC or KET treatment alone. The maximal [Ca^2+^]_i_ levels were achieved at 40 min after ALC and/or KET treatment. For this reason, [Ca^2+^]_i_ level change in different groups were further analyzed after ALC and/or KET treatment for 40 min. As shown in Fig. 5b, ALC (60 mM) and KET (100 μM) co-treatment significantly increased [Ca^2+^]_i_ levels at 40 min compared with ALC or KET treatment alone (*p* < 0.01). CNQX (50 μM) pretreatment could attenuate ALC- and ALC + KET-induced [Ca^2+^]_i_ level increase (*p* < 0.05); however, there was with no significant effect on KET-induced [Ca^2+^]_i_ level increase.

### ALC and KET co-treatment down-regulates CREB pathway-related protein expression and promotes apoptosis

**Figure 6:**
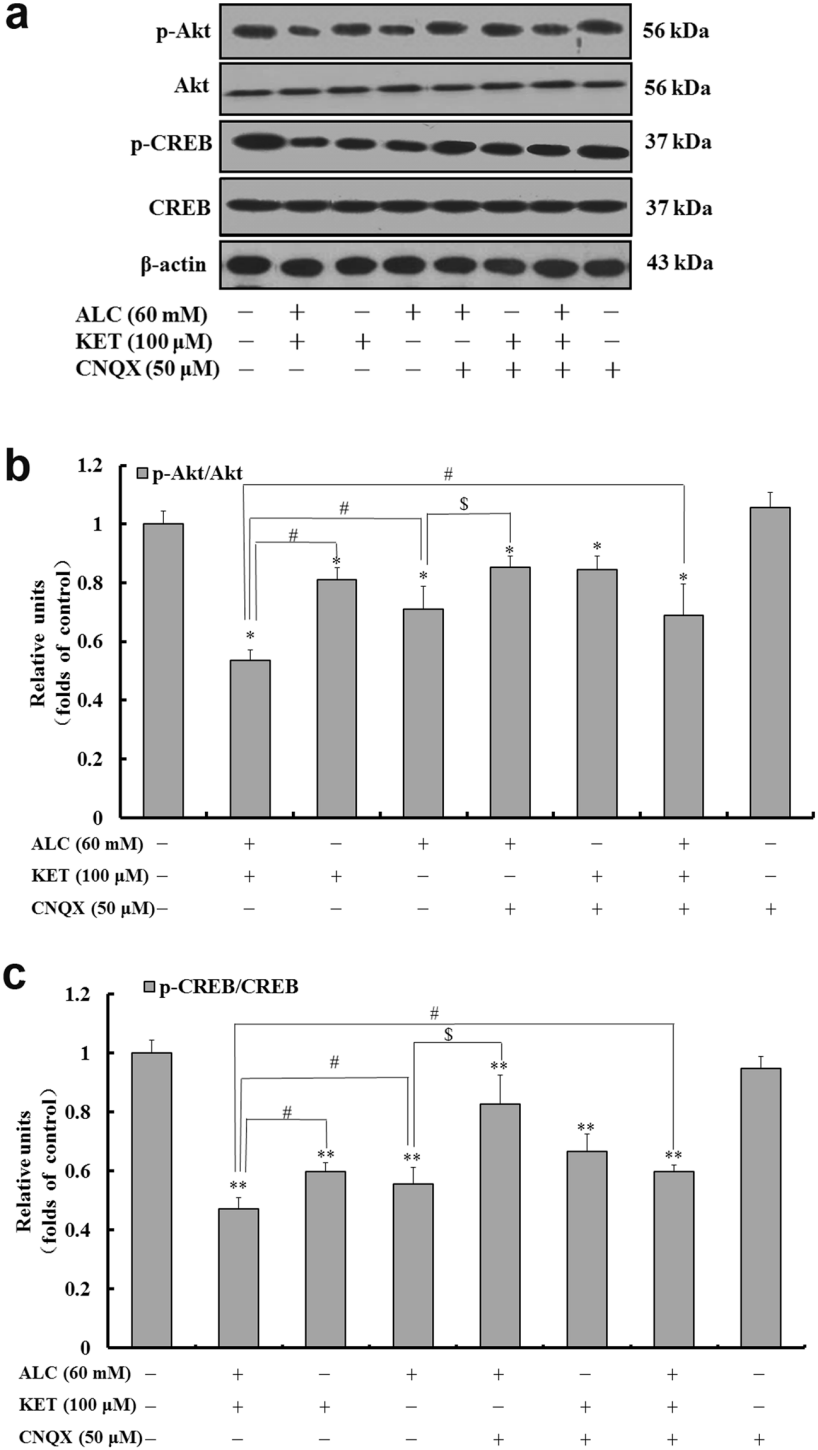
ALC potentiates KET-induced down-regulation of p-Akt and p-CREB expression. Representative immunoblots (**a**) and quantitative analysis of p-Akt/Akt (**b**) and p-CREB/CREB (**c**). The gels have been run under the same experimental conditions. The histogram in each panel indicated the relative band intensity generated from densitometric scans of three independent experiments. *p < 0.05, **p < 0.01 compared with the control group. ^#^ *p < *0.05 compared with the group treated with ALC and KET. ^$^ *p < *0.05 compared with the group treated with ALC.

**Figure 7:**
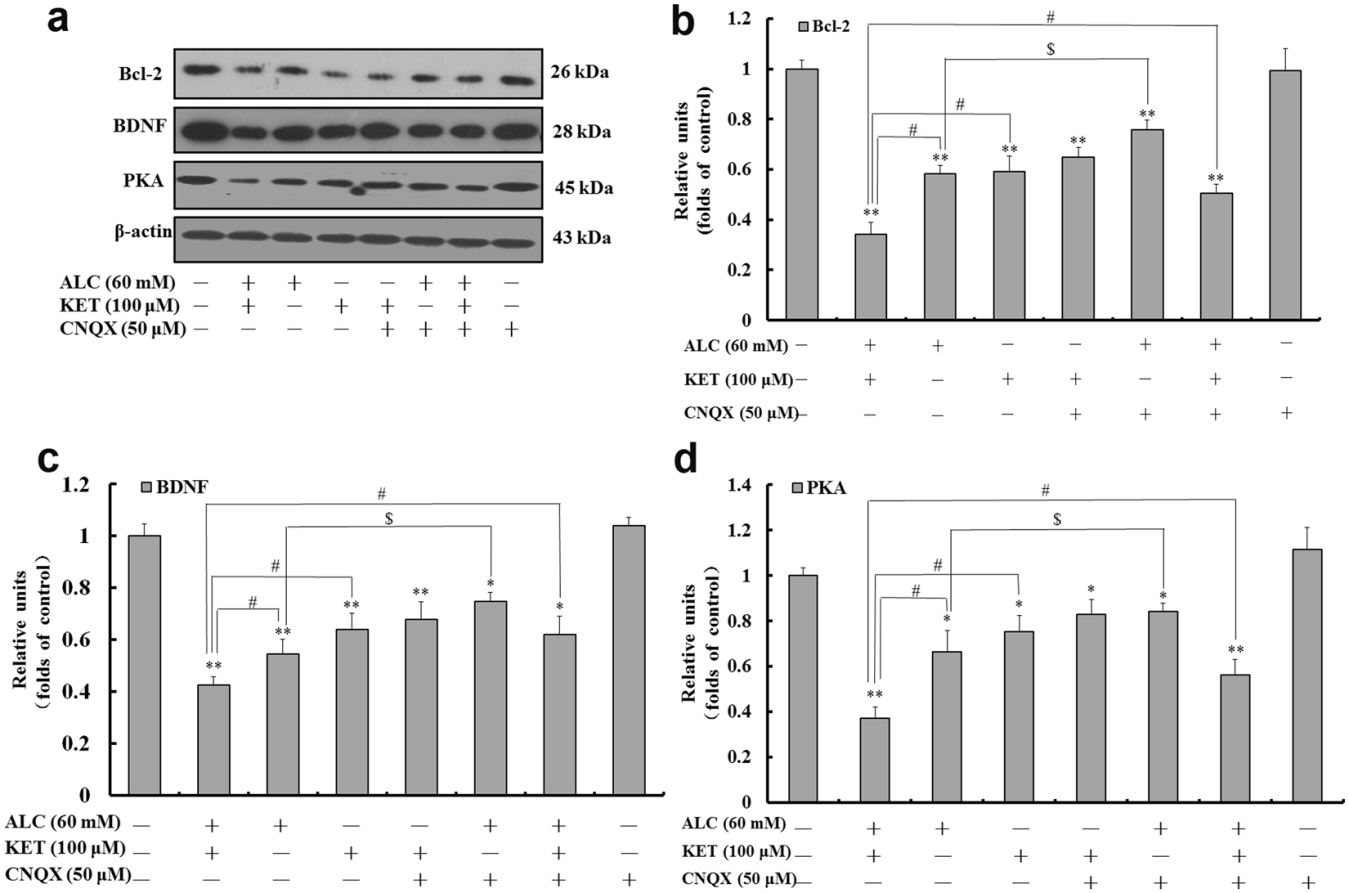
ALC potentiates KET-induced down-regulation of Bcl-2, BDNF and PKA expression. Representative immunoblots (**a**) and quantitative analysis of Bcl-2 (**b**), BDNF (**c**) and PKA (**d**). The gels have been run under the same experimental conditions. The histogram in each panel indicated the relative band intensity generated from densitometric scans of three independent experiments. *p < 0.05, **p < 0.01 compared with the control group. ^#^ *p < *0.05 compared with the group treated with ALC and KET. ^$^ *p < *0.05 compared with the group treated with ALC.

To further characterize the expression of CREB pathway-related protein, western blotting was performed. As shown in Figs 6 and 7, ALC or KET treatment for 24 h significantly down-regulated the expression of p-CREB, p-Akt, B cell lymphoma/lewkmia-2 (Bcl-2), BDNF and PKA. Co-exposure of ALC and KET for 24 h further down-regulated the expression of p-CREB, p-Akt, Bcl-2, BDNF and PKA compared with ALC or KET treatment alone (*p* < 0.05). CNQX (50 μM) pretreatment could attenuate ALC- and ALC + KET-induced down-regulation of p-CREB, p-Akt, Bcl-2, BDNF and PKA expression (*p* < 0.05).

**Figure 8:**
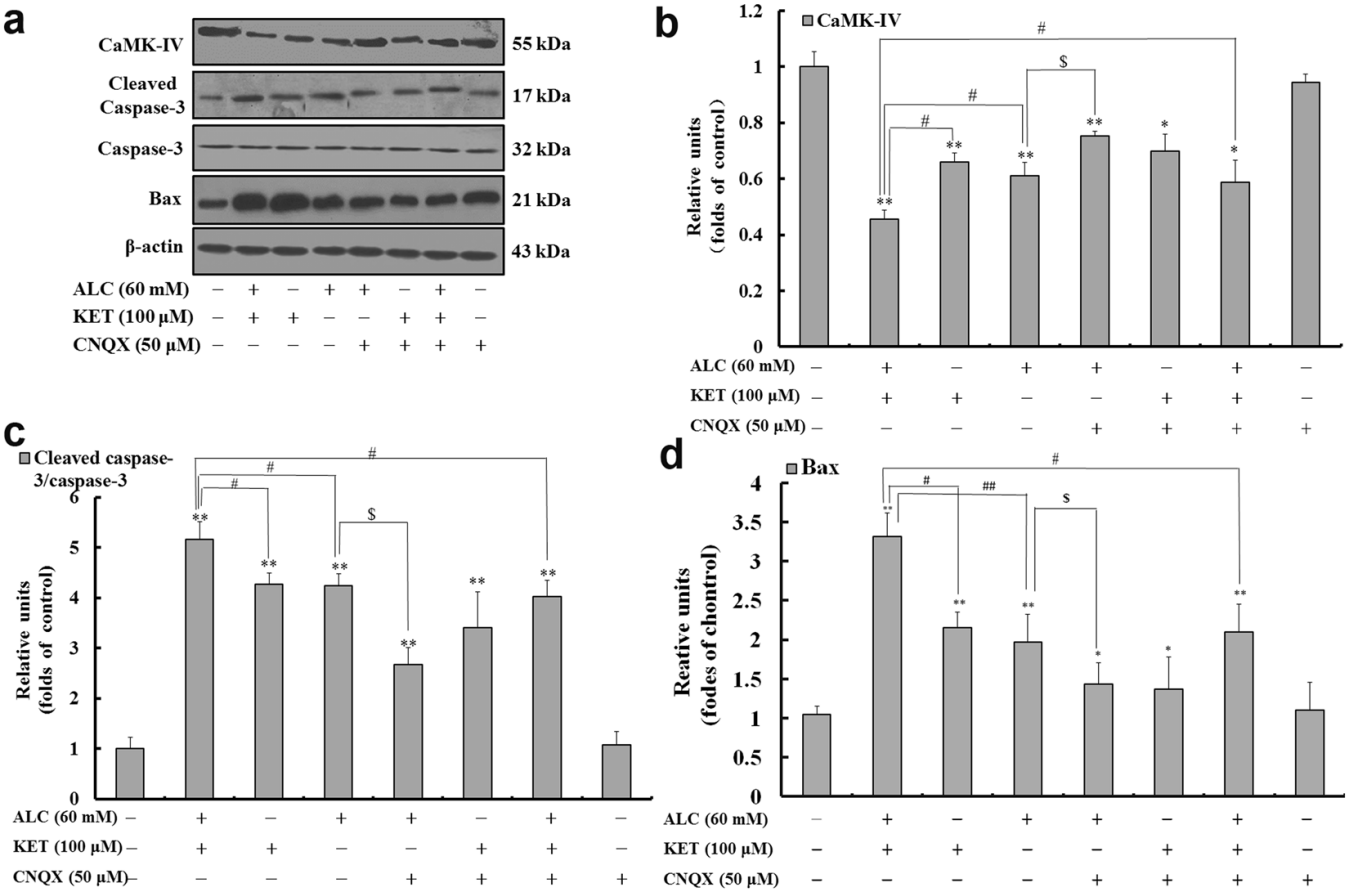
ALC potentiates KET-induced down-regulation of CaMK-IV and up-regulation of cleaved caspase-3 and Bax expression. Representative immunoblots (**a**) and quantitative analysis of CaMK-IV (**b**), cleaved caspase-3/caspase-3 (**c**) and Bax (**d**). The gels have been run under the same experimental conditions. The histogram in each panel indicated the relative band intensity generated from densitometric scans of three independent experiments. *p < 0.05, **p < 0.01 compared with the control group. ^#^ *p < *0.05, ^##^ *p < *0.01 compared with the group treated with ALC and KET. ^$^ *p < *0.05 compared with the group treated with ALC.

It was previously reported that intracellular Ca^2+^ could influence CREB via calmodulin-dependent kinase IV (CaMK-IV)^25^. As a result, we further explored the change of CaMK-IV expression. As shown in Fig. 8, ALC or KET treatment for 24 h significantly down-regulated the expression of CaMK-IV. Co-exposure of ALC and KET for 24 h further down-regulated the expression of CaMK-IV compared with ALC or KET treatment alone (*p* < 0.05). CNQX (50 μM) pretreatment could attenuate ALC- and ALC + KET-induced down-regulation of CaMK-IV expression (*p* < 0.05).

In addition, to further confirm the role of apoptosis in ALC and KET-induced neurotoxicity, the protein expression of caspase-3 and Bax was investigated by western blotting. As shown in Fig. 8, ALC or KET treatment significantly up-regulated the expression of cleaved caspase-3 and Bax compared with the control group (*p* < 0.01). Co-exposure of ALC and KET for 24 h further up-regulated the expression of cleaved caspase-3 and Bax compared with ALC or KET treatment alone (*p* < 0.05 or *p* < 0.01). CNQX (50 μM) pretreatment could lessen ALC- and ALC + KET-induced up-regulation of cleaved caspase-3 expression and Bax (*p* < 0.05).

### ALC and KET co-treatment down-regulates CREB and caspase-3 mRNA expression

**Figure 9:**
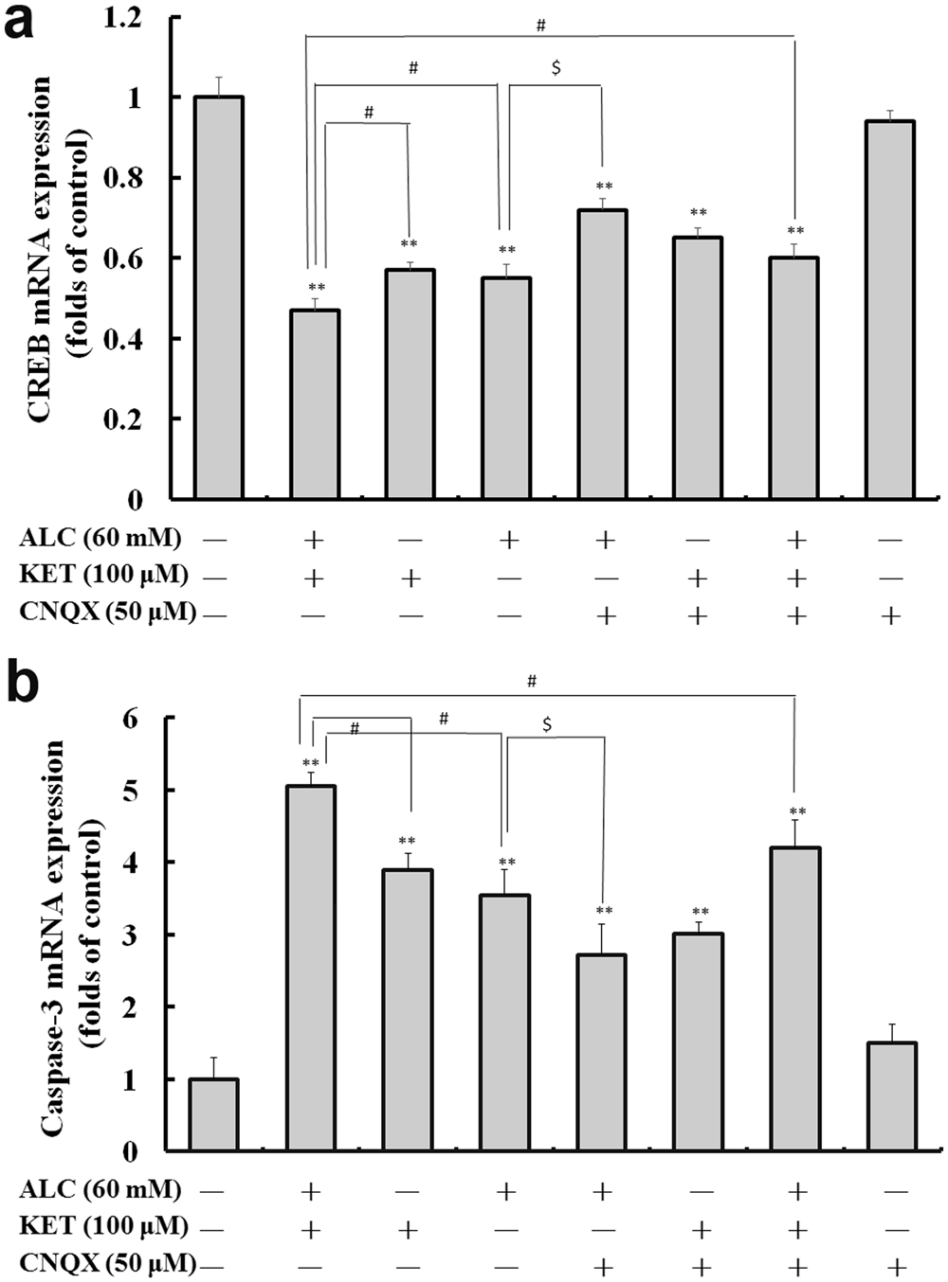
ALC potentiates KET-induced down-regulation of CREB (**a**) and caspase-3 (**b**) mRNA expression. **p < 0.01 compared with the control group. ^#^ *p < *0.05 compared with the group treated with ALC and KET. ^$^ *p < *0.05 compared with the group treated with ALC.

To further examine the key changes after ALC and/or KET treatment, CREB and caspase-3 mRNA expression was investigated by quantitative real-time PCR (qRT-PCR). As shown in Fig. 9a, ALC or KET treatment for 24 h significantly decreased CREB mRNA expression (*p* < 0.01). Co-exposure of ALC and KET for 24 h further decreased the mRNA expression of CREB compared with ALC or KET treatment alone (*p* < 0.05). CNQX (50 μM) pretreatment could attenuate the ALC- and ALC + KET-induced decrease of CREB mRNA expression (*p* < 0.05). In addition, as shown in Fig. 9b, ALC or KET treatment for 24 h significantly increased caspase-3 mRNA expression (*p* < 0.01). Co-exposure of ALC and KET for 24 h further increased the mRNA expression of caspase-3 compared with ALC or KET treatment alone (*p* < 0.05). CNQX (50 μM) pretreatment could reduce ALC- and ALC + KET-induced increase of caspase-3 mRNA expression (*p* < 0.05).

## Discussion

Mixed abuse of recreational drugs is very hazardous and has received increasing attention^26, 27^. In particular, mixed abuse of ALC and KET becomes a popular and specific combination of recreational drug abuse in adolescents at rave parties^19^. Since KET has a short acting effect, some abusers consume it many times per night, which will cause the accumulative dose per night per addict as high as several hundred milligrams. If the high dose of KET was accompanied with ALC, the toxicity will be significantly increased. There have been some reports of the lethality from mixed-drug intoxication involving ALC and KET^19, 20^. However, in most cases, the interplay between ALC and KET has not been fully characterized. In this study, PC12 cells were used to explore the neurotoxicity changes after exposure to ALC and/or KET. Our results demonstrated that ALC potentiated KET-induced neurotoxicity in PC12 cells. The cell viability was significantly decreased, ROS levels were significantly increased and the ultrastructural changes were more notable when KET was combined with ALC compared with ALC or KET treatment alone. Moreover, primary cultured cortical neurons were used to further examine the effect of ALC on KET-induced neurotoxicity. Our results provided the first evidence that the cell viability of primary cultured neurons was also significantly decreased when KET was combined with ALC compared with ALC or KET treatment alone, which was consistent with the results obtained with PC12 cells.

Previous studies have reported that KET has pro-apoptotic properties and induces neuroapoptosis in the developing brain and cultured neurons *in vitro* ^7, 28^. Our present study also verified that ALC or KET treatment alone did have pro-apoptotic properties in PC12 cells by AO-EB staining and FACScan flow cytometry, which was exacerbated when these use of two drugs were combined. These results indicate that KET in combination with ALC can cause a remarkable potentiation of their pro-apoptotic properties. Thus, our data suggest that the mixed use of ALC and KET may be more harmful for the CNS than their individual use.

ALC and KET can bind to and antagonize the NMDA receptor^29, 30^. It was reported that continuous blocking of NMDA receptors by NMDA receptor antagonists such as ALC or KET can cause a compensatory up-regulation of the NMDA receptor^31, 32^. This up-regulation can make cells bearing these receptors more vulnerable, which may be one of reasons for the neurotoxicity of KET and ALC since both of them are acting on the NMDA receptors^6, 33, 34^. Furthermore, the functions of NMDA and AMPA receptors are closely linked in drug addiction since they both belong to iGluRs [53]. In the present study, an interesting finding was that cell viability and apoptotic changes were attenuated when ALC alone or ALC + KET co-treatment of the cells were subsequently treated with CNQX, an AMPA/KA receptor antagonist. In contrast, KET-induced cell viability and apoptotic changes were not significantly altered by CNQX pretreatment. These results suggest that ALC- or ALC + KET-induced neurotoxicity was related to AMPA/KA receptor sensitivity. The activation of AMPA/KA receptors might further exacerbate ALC + KET-induced neurotoxicity. There was a greater association between the KET alone treatment-induced neurotoxicity and the compensatory up-regulation of the NMDA receptor than the AMPA/KA receptor sensitivity. In addition, we cannot exclude other possibilities since ALC and KET can also target to other receptors, which still need more investigation to elucidate.

Ca^2+^ plays a very important role in the process of neurotransmitter release and signal transduction after influx resulting from the activation of receptors and ion channels or as a second messenger^35, 36^. Even a small change in the level of [Ca^2+^]_i_ may produce major alterations in cellular activities^37^. Over-activation of the AMPA/KA receptors may induce a toxic accumulation of intracellular Ca^2+^, which may exacerbate the toxicity of PC12 cells. Therefore, in our present study, a fluorescent Ca^2+^ indicator Fura-4-AM, which diffuses across the cell membrane and is de-esterified by cellular esterases to yield Fura-4 free acid, was used to detect the changes of [Ca^2+^]_i_ levels. Our studies demonstrated that KET and/or ALC exposure caused time-dependent increase of [Ca^2+^]_i_ levels. The maximal increase of [Ca^2+^]_i_ levels was at 40 min after KET and/or ALC exposure. As a result, data in this time point were further analyzed. Our results verified that ALC and KET co-treatment further exacerbated Ca^2+^ accumulation. CNQX pretreatment attenuated ALC- and ALC + KET-induced Ca^2+^ accumulation, but with no significant effect on KET-induced Ca^2+^ accumulation, which indicates that Ca^2+^ accumulation after over-activation of the AMPA/KA receptors is involved in ALC + KET-induced neurotoxicity.

Akt, a downstream effector of the phosphoinositide-3-kinase (PI3K) pathway, plays an important role in regulating cell survival, growth, proliferation, transcription, metabolism and migration^38, 39^. Multiple apoptotic/survival regulating molecules are downstream substrates of Akt including CREB^40^. The phosphorylation of the transcription factor CREB by Akt on Ser133 results in its transcriptional activation. CREB is well known for its critical role in synaptic plasticity, neuronal development, and learning and memory function^41–43^. In this study, we addressed the important roles of Akt and CREB signaling molecules in ALC and KET-mediated neurotoxicity. Our findings demonstrated that ALC and/or KET decreased the phosphorylation of Akt and CREB, suggesting that ALC and KET might decrease the cell survival and promote apoptotic signaling by suppressing Akt and CREB activation. In addition, CNQX pretreatment could attenuate the suppression of Akt and CREB activation induced by ALC or ALC + KET treatment, which indicates that AMPA/KA receptor sensitivity is influenced by these processes.

In addition to the PI3K/Akt pathway, the other pathways that lead to CREB phosphorylation include the cAMP-PKA signaling pathway^44, 45^ and the Ca^2+^/CaMK-IV pathway^46^. In this study, we also investigated the changes of PKA and CaMK-IV. Our results revealed that PKA and CaMK-IV expression were significantly decreased after ALC and/or KET treatment, and CNQX pretreatment could attenuate these processes, which further demonstrated that AMPA/KA receptors are associated with the inhibition of the CREB-related cAMP-PKA pathway and the Ca^2+^/CaMK-IV pathway.

CREB promotes cell survival via a transcription-dependent mechanism, where it up-regulates the expression of anti-apoptotic genes such as Bcl-2^47^, which can prevent the release of cytochrome c to the cytosol and the subsequent activation of caspases^48^, thus inhibiting apoptosis. As a result, it is possible that the decrease in Bcl-2 gene expression and the increase in cleaved caspase-3 and Bax expression by ALC and KET co-treatment seen in our study was resulted from the inhibition of ALC and KET on the CREB-related pathways. In addition to Bcl-2, CREB also triggers the expression of other neuroprotective proteins such as BDNF^49, 50^. BDNF supports the survival of neurons, activates the growth and differentiation of new neurons and synapses and is known as an important synaptic modulator of synaptogenesis and synaptic plasticity^51–53^. In this study, BDNF expression was significantly down-regulated after ALC and/or KET treatment, and CNQX pretreatment could attenuate the down-regulation induced by ALC or ALC + KET treatment, which further indicated that ALC- or ALC + KET-induced neurotoxicity was associated with AMPA/KA receptor sensitivity.

Further qRT-PCR was conducted to explore the mRNA expression level changes for the main subtypes of AMPA and KA receptors after ALC and/or KET treatment in PC12 cells. Our data showed that the mRNA expression level of GluR2, one of main subtypes of AMPA receptors, was decreased after ALC and/or KET treatment (Supplementary Fig. 1a). However, the mRNA expression levels of KA2 (Supplementary Fig. 1b) and GluR6 (Supplementary Fig. 1c), the main subtypes of KA receptors, were significantly increased after ALC and/or KET treatment. These additional data indicate that the neurotoxicity of ALC and KET combination might be related with the activation of KA receptors and the inhibition of AMPA receptors might also be involved in this process. Further studies are still needed to explore the involvement of these receptors in ALC and KET-induced neurotoxicity.

**Figure 10:**
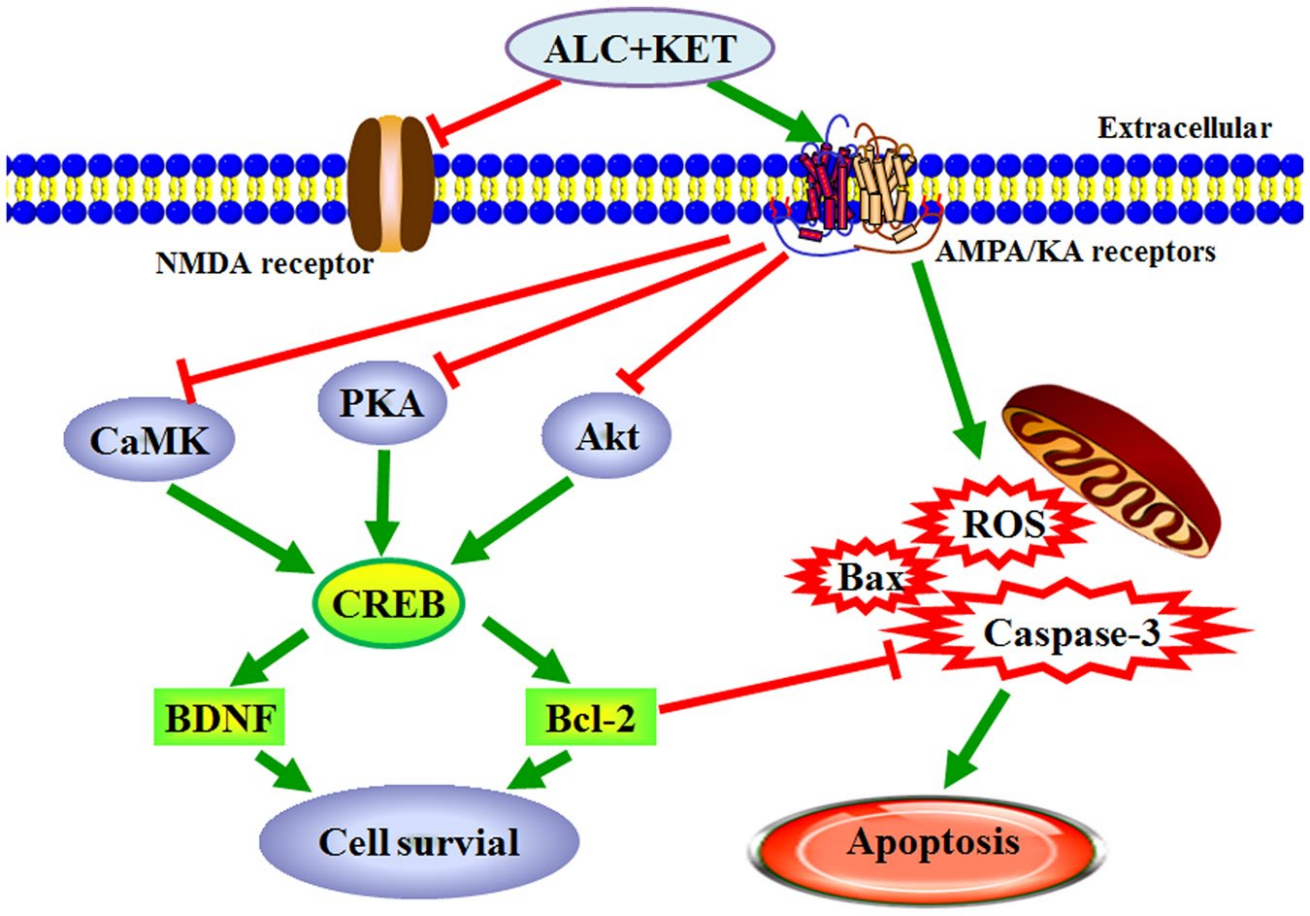
Mechanism summary of the ALC + KET-induced neurotoxicity.

Taken together, our study provides new evidence that co-treatment of ALC in PC12 cells and primary cultured neurons potentiates the neurotoxicity of KET. This phenomenon occurs through the inhibition of Akt-, PKA- and CaMK-IV-mediated CREB signaling pathway, and then induced apoptosis of PC12 cells (Fig. 10). An *in vivo* study using adolescent mice or rats would be necessary to further characterize the nature and degree of neurotoxicity in animals.

## Materials and Methods

### Reagents

KET hydrochloride was obtained from Gutian Medical Inc. (Fujian, China). ALC was purchased from Beijing Red Star Co. (Beijing, China). CNQX and Fura-4-AM were purchased from Sigma (USA). Dulbecco’s modified eagle medium (DMEM)/F12, B27 supplement and fetal bovine serum (FBS) were purchased from Gibco (NY, USA). MTT was from Ameresco (USA). βШ-tubulin was from Sigma (USA). Cytosine-β-D-arabinoside (Ara-C), DCFH-DA and DAPI were from Beyotime (Nanjing, China). Hoechst 33258, propidium iodide (PI) and AO-EB were from Solarbio (Beijing, China). Trizol reagent and HiFi-script cDNA Kit were purchased from CWBIO (Beijing, China). UltraSYBR Mixture (low ROX) was purchased from LEWEITECH (Shijiazhuang, China). Primary antibodies against Akt, p-Akt, CREB, p-CREB, PKA, CaMK-IV, Bcl-2, cleaved caspase-3 and horseradish peroxidase (HRP)-conjugated secondary antibodies (goat-anti-rabbit and goat-anti-mouse) were purchased from Bioss (Beijing, China). Rabbit anti-caspase-3 and BDNF IgG and mouse anti-β-actin IgG were purchased from ZSGB-BIO (Beijing, China). Primary antibody against Bax was from Proteintech (USA). Enhanced chemiluminescence was obtained from Amersham Biosciences (England, UK).

### PC12 cell cultures

PC12 cell line was obtained from Shanghai cell bank of Chinese Academy of Sciences. The cells were cultured in DMEM containing 10% FBS, 100 U/ml streptomycin and 100 U/ml penicillin at 37 °C in humidified atmosphere with 5% CO_2_. The culture medium was replaced every 48 h, and cell cultures were passaged at a ratio of 1:5 every 4 days.

### Primary culture of rat cortical neuronal cells

Primary cultured neurons were prepared as previously described^54^. Animal procedures were conducted in accordance with the National Institutes of Health guide for the care and use of Laboratory animals (NIH Publications No. 8023, revised 1978) and approved by Ethics Committee of Shenyang Pharmaceutical University. Briefly, cerebral cortex of neonatal Sprague-Dawley rats (postnatal day 1) was dissected and placed in ice-cold DMEM, then mechanically dissociated and digested with trypsin. The cells (10^6^ cells/ml) were distributed and grown in DMEM/F12 with 15% FBS, 100 U/ml penicillin, 100 U/ml streptomycin sulfates at 37 °C in a humidified 5% CO_2_/95% air atmosphere. Neuron culture medium was replaced by serum-free DMEM/F12 + 2% B27 supplement at 48 hours, subsequently half of the culture medium was replaced every 48 hours. On the 4th day, cultures were fed with medium containing Ara-C (2.5 μg/ml) for 48 hours to prevent glial cell growth. The neurons cultured for 6 days were ready for the experiments.

### Measurement of cell viability

Cell viability was measured by the MTT assay according to our previous report^54^. Briefly, the medium was incubated with 10 μl of 5 mg/ml MTT solution before the end of the experiment for 4 h at 37 °C. Then the culture medium with MTT was removed and 200 μl dimethyl sulfoxide was added to each well to dissolve the formazan. Absorbance was measured at 492 nm with microtiter plate reader (ThermoFisher, Shanghai, China). The absorbance of the control (A_control_) was set as 100% viability. The absorbance of treated cells (A_experimental_) was related and normalized to that of the control-cells. A_background_ is the absorbance of culture medium plus MTT in the absence of cells. The viability of the cells was defined as: Cell viability = [(A_experimental_ − A_background_)/(A_control_ − A_background_)] × 100%.

### Intracellular ROS measurement

ROS level was detected by DCFH-DA staining. DCFH-DA is a stable, nonpolar compound which readily diffuses into cells and is hydrolyzed by intracellular esterase to yield the DCFH, which is trapped into the cells. ROS produced by the cells oxidize DCHF to highly fluorescent compound 2′,7′-dichlorofluorescein (DCF). Thus, the fluorescence intensity of DCF is directly ratio to the amount of ROS produced by the cells. After ALC and/or KET treatments for 24 h, cells were washed twice with PBS. Then they were loaded with DCFH-DA (10 μM) and incubated at 37 °C for 30 min. The intensities of fluorescence were detected by fluorescence microscopy and photographed (Olympus TH4200). Final results were shown as percentage of control group.

### Transmission electron microscopy

Transmission electron microscopy was used to study the ultrastructural changes induced by ALC and KET treatment. The collected cells were fixed with 3% glutaraldehyde and post-fixed with 1%OsO4, then embedded and sectioned. The sections were stained with uranyl acetate and lead citrate, and examined by an H-7650 transmission electron microscope (Hitachi, Tokyo, Japan).

### Immunofluorescence staining of the primary cultured cells

The neuronal cells were fixed with 4% para-formaldehyde for 30 min, then rinsed with PBS and permeabilized with 0.2% Triton X-100 in PBS for 20 min at room temperature. After that, cells were incubated with bovine serum albumin in PBS for 20 min at room temperature for blockade of non-specific binding sites. After incubation for 16–18 h at 4 °C in humid chamber with rabbit anti-βШ-tubulin (1:100), FITC labeled goat anti-rabbit IgG (1:100) was added to incubate for another 2 h at 37 °C. The nuclei were stained with DAPI for 20 min. Thereafter, the plates were rinsed with PBS and photographed under fluorescence microscopy (Olympus TH4200).

### Nuclear morphological analysis

Apoptosis changes in the nuclear chromatin of PC12 cells were tested by DAPI staining. Briefly, the cells were seeded in 24-well plates for 24 h, then treated with different drugs for 24 h. After that, the cells were washed with PBS and then incubated with DAPI for 10 min. After being washed with PBS, the cells were observed and photographed using a fluorescence microscope (Olympus, Tokyo, Japan) and quantified by ImageJ 1.44 software.

### AO-EB double staining

To detect apoptosis, cells were stained with AO-EB. AO could pass through intact cell membrane and color DNA as green fluorescence, whereas EB passes through broken cell membrane coloring DNA as orange fluorescence. Morphological difference between normal cells and apoptotic cells could be observed under a fluorescence microscope. For the AO/EB procedure, PC12 cells seeded in 24-well plates (1 × 10^6^ cells/well) were grown at 37 °C, 5% CO_2_. After 24 h treatments of different drugs, the cells were washed with PBS and stained with AO-EB (100–100 μg/ml) solution. Cell morphology was observed by fluorescence microscope (Olympus, Tokyo, Japan).

### Apoptosis analysis by FACScan flow cytometry

After the treatment of different drugs for 24 h, the cells were collected and fixed in ice-cold 70% ethanol for 12 h, then stained with 50 μg/ml PI on 4°C for 1 h. After that, the samples were analyzed by FACScan flow cytometry (Becton–Dickinson, Franklin Lakes, NJ, USA).

### Determination of [Ca^2+^]_i_ levels

[Ca^2+^]_i_ was determined by fluorometric analysis using molecular probes Fura-4-AM^37^. Briefly, PC12 cells were loaded with 2 μM Fura-4-AM and incubated for 30 min at 37 °C in 5% CO_2_. Unbound Fura-4-AM was removed by rinsing the cells twice with PBS. Then, the cells were treated with different drugs. [Ca^2+^]_i_ fluorescence was measured by fluorescence microscope (Olympus, Tokyo, Japan) every 10 min for 60 min. The concentration of intracellular [Ca^2+^]_i_ was calculated using the following formula: [Ca^2+^]_i_ = K_d_ × (F_o_ − F_min_)/(F_max_ − F_o_), where K_d_ is the dissociation constant of Fura-4-AM calcium complex, and its value is 224 nmol/L, and F_o_ is the measured fluorescence optical density value of different time points, F_max_ and F_min_ are fluorescence optical density values of calcium saturation and absence, respectively. The results are expressed as nanomoles of calcium.

### Quantitative Real-Time PCR

Gene expression levels of CREB and caspase-3 were quantified by qRT-PCR. RNA was extracted with Trizol reagent according to the manufacturer’s instructions. First-strand cDNA was synthesized using HiFi-script cDNA Kit on an AlphaTM Unit Block Assembly for DNA Engine Systems (Germany). Amplification was achieved with UltraSYBR Mixture (low ROX), forward and reverse primers on a Stratagene M × 3000 P (Aligent, Germany). The primer sequences were as follows: CREB, forward primer: 5′-TCAGCCGGGTACTACCATTC-3′ and reverse primer: 5′-TCTCTTGCTGCCTCCCTG-3′; Caspase-3, forward primer: 5′-GTGTCCATGCTCACGAAAGA-3′ and reverse primer: 5′-CCAGGAGGACCGTCAGATTA-3′; and GAPDH, forward primer: 5′-GTATGACTCCACTCACGGCAA-3′ and reverse primer: 5′-CACCAGTAGACTCCACGACA-3′.

### Western blotting analysis

Protein preparation and immunoblot analyses were carried out according to previous procedures^55^. In brief, the protein concentration was measured using a protein assay kit (Bio-Rad, USA). The protein lysates were separated by sodium dodecyl sulfate-polyacrylamide gel electrophoresis (SDS-PAGE) and transferred to a nitrocellulose membrane (Millipore Corporation, USA). The membranes were probed with Akt (1: 500), p-Akt (1: 300), CREB (1: 300), p-CREB (1: 300), PKA (1: 300), BDNF (1: 300), CaMK-IV (1: 300), Bcl-2 (1: 300), cleaved caspase-3 (1: 300), caspase-3 (1: 500) and Bax (1: 800) at 4 °C overnight and then incubated with a horseradish peroxidase (HRP)-conjugated secondary antibodies (1: 800) at 37 °C for 2 h. Proteins were visualized using enhanced chemiluminescence. Experiments were repeated at least three times. Densitometry analysis was done using ImageJ 1.44 software.

### Statistical analysis

Data from at least three independent experiments were expressed as the mean ± SD. Statistical analysis was performed by the one-way analysis of variance (ANOVA) followed by Newman–Keuls’ test using SPSS software 19.0. The values of *p* < 0.05 were considered as significant, *p* < 0.01 more significant compared to the indicated experimental group.

## Electronic supplementary material

Supplementary information
