# Supplementary material for: Alcohol amplifies ketamine-induced apoptosis in primary cultured cortical neurons and PC12 cells through down-regulating CREB-related signaling pathways
Source: Sci Rep. 2017 Sep 5;7:10523. doi: 10.1038/s41598-017-10868-z (PMC5585325; doi:10.1038/s41598-017-10868-z)
Supplement: Supplementary file 1 — Supplementary information [file 41598_2017_10868_MOESM1_ESM.pdf]

Supplementary information

Alcohol amplifies ketamine-induced apoptosis in primary cultured cortical neurons and PC12 cells through down-regulating CREB-related signaling pathways

Daiying Zuo*, Feng Sun, Jiahui Cui, Yumiao Liu, Zi Liu, Xuejiao Zhou, Zengqiang Li, Yingliang Wu*

Department of pharmacology, Shenyang Pharmaceutical University, 103 Wenhua Road, Shenyang, Liaoning 110016, PR China

*Correspondence and requests for materials should be addressed to Y.W. (email: yingliang_wu@hotmail.com) or D.Z. (email: zuodaiying@163.com)

Supplementary figure

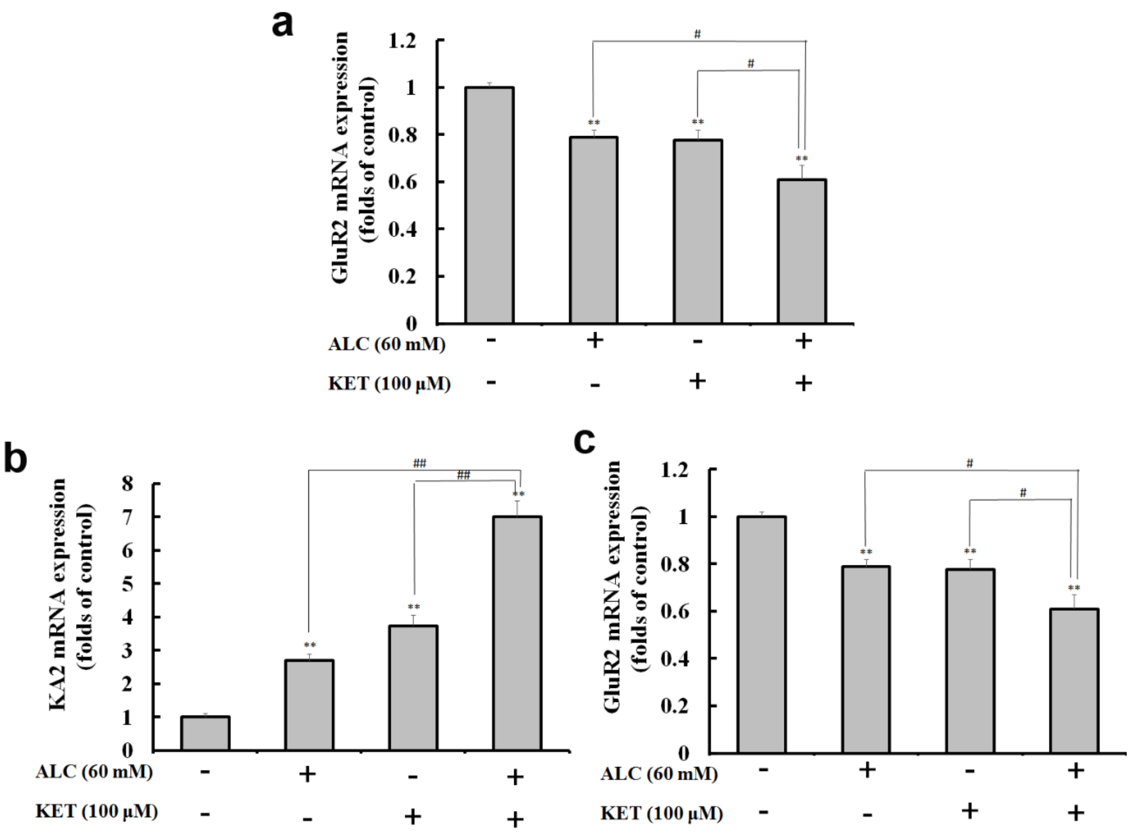

Supplementary Figure 1. The mRNA expression level changes for the main subtypes of AMPA and KA receptors. ALC and/or KET treatment decreased the mRNA expression level of GluR2 (a), one of main subtypes of AMPA receptors, and increased the mRNA expression levels of KA2 (b) and GluR6 (c), the main subtypes of KA receptors. $^{**}p < 0.01$ compared with the control group. $^{\#}p < 0.05$, $^{\#\#}p < 0.01$ compared with the group treated with ALC and KET.

Supplementary method

Quantitative Real-Time PCR. Gene expression levels of GluR2, GluR6 and KA2 were quantified by qRT-PCR. RNA was extracted with Trizol reagent according to the manufacturer's instructions. First-strand cDNA was synthesized using HiFi-script cDNA Kit on an AlphaTM Unit Block Assembly for DNA Engine Systems (Germany). Amplification was achieved with UltraSYBR Mixture (low ROX), forward and reverse primers on a Stratagene M × 3000P (Aligent, Germany). The primer sequences were as

follows: GluR2, forward primer: 5’-TTCCTGGTCAGCAGATTTAGCC-3’ and reverse primer: 5’-TGGGAGACACCATCCTCTCTACAG-3’; GluR6, forward primer: 5’-GGTATAATCCACACCCTTGCAACC-3’ and reverse primer: 5’-TGACTCCATTAAGAAAGCATAATCGGA-3’; KA2, forward primer: 5’-TCGCCCGCGTCCTCAACTCC-3’ and reverse primer: 5’-CACCGACACCTCCTCGGACT-3’; and GAPDH, forward primer: 5’-GTATGACTCCACTCACGGCAA-3’ and reverse primer: 5’-CACCAGTAGACTCCACGACA-3’.
